# Comparative Evaluation of Diagnosis and Treatment Plan Given by Pediatric Dentists and Generated by ChatGPT: A Cross-Sectional Pilot Study

**DOI:** 10.7759/cureus.88505

**Published:** 2025-07-22

**Authors:** Shilpa Naik, Rucha Bhise, Sanjana R Kodical, Shirin Chavan, Sonal S Mali, Nidhi Shetiya

**Affiliations:** 1 Pedodontics and Preventive Dentistry, DY Patil Deemed to be University School of Dentistry, Navi Mumbai, IND; 2 Pediatric and Preventive Dentistry, DY Patil Deemed to be University School of Dentistry, Navi Mumbai, IND; 3 Pediatric Dentistry, DY Patil Deemed to be University School of Dentistry, Navi Mumbai, IND

**Keywords:** artificial intelligence, artificial intellingence in dentistry, diagnostic accuracy, pediatric dentistry, treatment planning

## Abstract

Background: Artificial intelligence (AI) is rapidly transforming healthcare, including dental specialties such as pediatric dentistry. Chat generative pre-trained transformer (ChatGPT), an AI language model, has demonstrated potential in patient communication and academic writing, but its clinical utility in diagnostic and treatment planning contexts remains underexplored.

Aim: To evaluate the diagnostic accuracy and treatment planning capabilities of ChatGPT in comparison with those of experienced pediatric dentists.

Materials and Methods: A cross-sectional pilot study was conducted involving 10 children aged 6-12 years. Standardized case scenarios were developed using clinical findings, medical history, and radiographs. Four pediatric dentists independently assessed each case and provided diagnoses and treatment plans. The same case scenarios were input into ChatGPT (v3.5), and its outputs were reviewed by a fifth pediatric dentist. A senior pediatric dentist blinded to the source validated all responses. Accuracy was compared, and inter-rater agreement was assessed using Cohen’s kappa statistics.

Results: ChatGPT achieved an 80% accuracy rate in diagnosis and treatment planning, equivalent to two pediatric dentists and slightly lower than the 90% accuracy seen in the other two. Statistical analysis revealed no significant difference between the performance of ChatGPT and the pediatric dentists (p = 0.926). Cohen’s kappa indicated almost perfect agreement with two dentists (κ = 1.00) and moderate agreement with the others (κ = 0.615).

Conclusion: ChatGPT demonstrated diagnostic accuracy comparable to pediatric dentists and may serve as a valuable adjunct in clinical pediatric dentistry. Larger studies are needed to confirm these findings and guide responsible integration of AI tools.

## Introduction

Chat generative pre-trained transformer (ChatGPT) is an advanced artificial intelligence (AI)-driven language model that has been designed to process and generate human-like responses based on extensive datasets. It employs deep-learning and machine-learning algorithms to enhance computational linguistics, communication competence, and responsiveness [1]. By utilizing text-based interfaces, ChatGPT can engage in interactive dialogues, providing contextually relevant information across a wide range of topics, including healthcare and dentistry. AI-driven models like ChatGPT have the potential to transform the way healthcare professionals access and deliver information, with growing interest in their applications for diagnostics, treatment planning, and patient education [2,3].

In recent years, AI has made significant strides in various medical and dental applications, particularly in diagnostic imaging, disease detection, and patient management. The field of dentistry has seen advancements in AI-based systems capable of identifying caries, assessing periodontal health, and aiding in orthodontic treatment planning [4,5]. The use of deep-learning algorithms, such as convolutional neural networks (CNNs), has demonstrated a high degree of accuracy in detecting dental pathologies, often rivaling expert clinical assessments [6]. Given these advancements, it is essential to compare the accuracy and reliability of diagnoses provided by AI tools like ChatGPT with those made by pediatric dentists to determine their potential role in clinical decision-making.

Pediatric dentistry presents unique challenges that AI-driven tools may help address. Young patients often experience dental anxiety, making behavior management a critical aspect of pediatric dental care. AI-powered technologies, including virtual reality (VR) and augmented reality (AR), are increasingly being explored to create engaging and interactive experiences that help alleviate anxiety and improve patient cooperation [7,8]. Additionally, AI can contribute to personalized oral health recommendations by analyzing individual risk factors and providing tailored preventive care strategies [9]. Given the increasing reliance on AI in various domains of dentistry, it is important to assess the feasibility of integrating AI-assisted tools into pediatric dental practice to determine their practical utility in real-world clinical settings.

Beyond diagnosis, effective treatment planning is a cornerstone of pediatric dental care, ensuring optimal patient outcomes [10]. AI models have been utilized in detecting dental anomalies, caries risk assessment, and periodontal disease detection in children [6,10]. A recent study has demonstrated that CNNs can accurately identify plaque accumulation on primary teeth, suggesting that AI can complement clinical examinations and facilitate early interventions [11]. While these developments are promising, the extent to which AI-generated treatment plans align with those formulated by pediatric dentists remains largely unexplored. To establish the credibility of AI in pediatric dentistry, it is crucial to evaluate the degree of agreement between human experts and AI-generated treatment recommendations.

Despite the potential advantages of AI in pediatric dentistry, its application in this field remains in its early stages, requiring further validation. While ChatGPT has been explored as a tool for patient education and academic content generation, its ability to provide clinical recommendations in pediatric dentistry has not been extensively studied. As AI continues to evolve, it is imperative to determine whether such models can serve as a reliable adjunct to traditional clinical expertise. Therefore, this study aims to assess the quality of information generated by ChatGPT and compare it with the diagnosis and treatment plans given by pediatric dentists. By doing so, this study will provide insight into the role AI can play in pediatric dental care, its limitations, and its future potential in clinical practice.

## Materials and methods

The present study was designed as a cross-sectional pilot study aimed at evaluating the diagnostic accuracy and treatment planning capabilities of ChatGPT in comparison to human pediatric dentists. The study was conducted in a Pediatric and Preventive Dentistry outpatient department (OPD) over a period of one week, from March 4 to March 9, 2024. 

Ethical approval

Prior to the commencement of the study, the study protocol was approved by the institutional Research and Ethical Board, DY Patil University School of Dentistry, Navi Mumbai (Reference Letter No. IREB/2025/PEDO/02, dated June 18, 2025). Informed consent was obtained from the parents and verbal consent was obtained from the children.

Participant selection

Inclusion criteria required that participants be within the specified age range of 6-12 years and present with a clearly defined chief complaint related to teeth that required clinical diagnosis and treatment planning. Only children exhibiting cooperative behavior that allowed for effective clinical examination were included in the study. Additionally, only those without any known systemic illnesses, syndromic conditions, or special healthcare needs were considered eligible. Children who were medically compromised, exhibited uncooperative behavior that hindered clinical evaluation, or required multidisciplinary care such as surgical or orthodontic intervention were excluded to maintain uniformity in case complexity and ensure the reliability of diagnostic assessment.

Data collection and case scenario development

Each patient’s clinical case was documented in a standardized format, including chief complaint, relevant history, clinical findings, and radiographs (if available). To maintain confidentiality and ethical integrity, all patient data were anonymized and de-identified prior to input into ChatGPT and prior to review by participating clinicians. No personally identifiable information was used at any stage of the data processing or analysis. A structured data-collection process was employed to ensure the reliability and standardization of case documentation, assessment, and evaluation. Standardized case presentation formats, as recommended in recent dental AI research studies, were adopted to maintain consistency and minimize bias in case documentation [12].

Assessment by pediatric dentists

Following the case documentation, four experienced pediatric dentists (Observers I-IV) independently assessed each case. They provided their diagnoses and formulated treatment plans without external influence or discussion among themselves. This step ensured an unbiased expert-driven comparison of diagnostic accuracy and treatment planning.

ChatGPT evaluation and blinded outcome assessment

The same documented case scenarios were input into OpenAI’s ChatGPT (version 3.5) using a structured query format designed to replicate the diagnostic approach of a practicing clinician. A sample prompt is as follows:

“A 7-year-old child presents with pain in the lower right back tooth for three days. On examination, deep dental caries is observed in tooth 85 with tenderness on percussion. The child is cooperative and has no relevant medical history. What is the most likely diagnosis and appropriate treatment plan in a pediatric dental setting?”

The AI-generated responses included diagnoses and treatment recommendations, which were then evaluated for their clinical validity. A fifth pediatric dentist (Observer V) reviewed the responses generated by ChatGPT to assess their coherence and alignment with standard pediatric dental guidelines. To ensure an objective evaluation, a senior pediatric dentist with over ten years of clinical experience independently reviewed and validated all responses-both those provided by human dentists and those generated by ChatGPT. This assessment was conducted in a blinded manner, meaning the reviewer was unaware of the source of each diagnosis and treatment plan.

Statistical analysis

Given the relatively small sample size (n = 10), descriptive statistical methods were employed to summarize key findings. Statistical analysis was performed using Statistical Package for Social Sciences Software (SPSS v23, IBM). Differences in diagnostic/treatment decisions between the pediatricians and the AI tool (ChatGPT) were assessed using Fisher’s Exact Test. Inter-rater agreement between the pediatricians and ChatGPT was evaluated using Cohen’s kappa statistic. A p-value less than 0.05 was considered statistically significant.

## Results

Study population

The study included 10 pediatric patients reporting to the Pediatric and Preventive Dentistry OPD with various dental complaints who satisfied the eligibility criteria. The study aimed to compare the diagnostic accuracy and treatment planning capability of ChatGPT AI with that of four experienced pediatric dentists.

Diagnosis and treatment accuracy

Each patient case was assessed independently by four pediatric dentists and ChatGPT AI. The frequency and percentage of correct and incorrect diagnoses/treatments provided by each rater are summarized in Table 1.

**Table 1:** Diagnosis/treatment proposed by pediatric dentists and ChatGPT AI ChatGPT: Chat generative pre-trained transformer; AI: Artificial intelligence

By paediatricians		Frequency	Percentage
Pediatrician 1	Incorrect diagnosis/treatment	2	20.0
	Correct diagnosis/treatment	8	80.0
Pediatrician 2	Incorrect diagnosis/treatment	2	20.0
	Correct diagnosis/treatment	8	80.0
Pediatrician 3	Incorrect diagnosis/treatment	1	10.0
	Correct diagnosis/treatment	9	90.0
Pediatrician 4	Incorrect diagnosis/treatment	1	10.0
	Correct diagnosis /treatment	9	90.0
ChatGPT AI	Incorrect diagnosis/treatment	2	20.0
	Correct diagnosis/treatment	8	80.0

**Figure 1:**
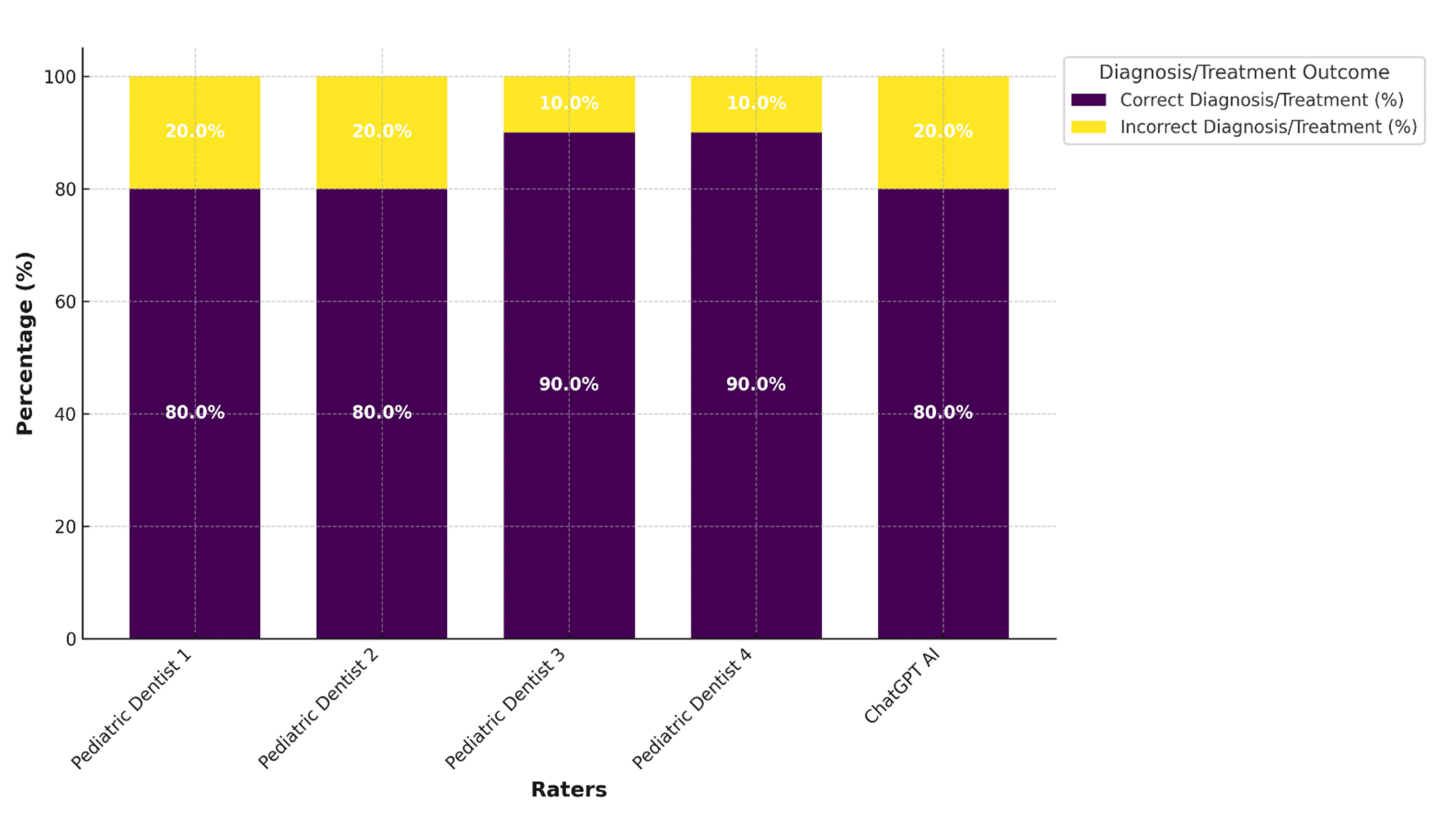
Percentage of correct and incorrect diagnoses/treatment plans provided by the raters and ChatGPT ChatGPT: Chat generative pre-trained transformer

Figure 1 illustrates the percentage of correct and incorrect diagnoses/treatment plans provided by the pediatric dentists and ChatGPT AI. The graph visually demonstrates the similarities in accuracy rates among the raters, further supporting the conclusion that AI-generated recommendations align closely with expert decisions of the pediatric dentists.

Inter-rater reliability

To assess the level of agreement between the pediatric dentists and ChatGPT AI, Cohen’s kappa coefficient was used. The results are summarized in Table 2.

**Table 2:** Inter-Rater Reliability Using Kappa Statistics * Cohen’s kappa values between 0 and .20 indicate no agreement beyond chance, with only 0–4% of the data being reliable, while values between .21 and .39 suggest minimal agreement, where 4–15% of the data is reliable. A kappa value between .40 and .59 reflects weak agreement, with 15–35% of the data being reliable, and values between .60 and .79 show moderate agreement, with 35–63% of the data being reliable. Strong agreement is observed with kappa values between .80 and .90, where 64–81% of the data is reliable, and values above .90 represent almost perfect agreement, with 82–100% of the data being reliable. ChatGPT: Chat generative pre-trained transformer; AI: Artificial intelligence

Rater	Kappa statistics	Significance (p)	Interpretation*
Rater 1 with ChatGPT AI	1.00	0.002	Almost perfect agreement
Rater 2 with ChatGPT AI	1.00	0.002	Almost perfect agreement
Rater 3 with ChatGPT AI	0.615	0.035	Moderate agreement
Rater 4 with ChatGPT AI	0.615	0.035	Moderate agreement

The kappa statistics indicate almost perfect agreement between ChatGPT AI and Observers I and II, while a moderate agreement was observed with Observers III and IV. These findings highlight the potential reliability of AI in assisting with diagnostic decision-making in pediatric dentistry. Overall, the results suggest that ChatGPT AI demonstrates comparable diagnostic and treatment planning accuracy to experienced pediatric dentists, reinforcing its potential as a valuable tool in pediatric dental practice.

## Discussion

The findings of this study provide valuable insights into the potential role of AI, particularly ChatGPT, in pediatric dental diagnosis and treatment planning. AI has been increasingly integrated into various healthcare domains, with its application in dentistry gaining significant attention in recent years [4,5,9]. The ability of AI models to process vast amounts of data, recognize patterns, and provide structured responses has made them promising tools for clinical decision-making. In pediatric dentistry, where accurate diagnosis and appropriate treatment planning are crucial for ensuring long-term oral health, the evaluation of capabilities of AI against human expertise is particularly relevant. The results demonstrated that ChatGPT exhibited an 80% accuracy rate in diagnosing and providing treatment recommendations, aligning closely with two of the four pediatric dentists who also had an accuracy rate of 80%. The remaining two pediatric dentists showed a slightly higher accuracy of 90%. This suggests that AI models like ChatGPT can perform comparably to experienced clinicians in specific diagnostic and treatment planning scenarios. The absence of a statistically significant difference between the diagnostic accuracy of ChatGPT and that of human pediatric dentists further reinforces the potential of AI as an adjunctive tool in pediatric dental practice. However, it is important to consider that AI models are trained on pre-existing data and do not possess real-time clinical experience, which may limit their ability to account for subtle variations in patient presentations that a human clinician would recognize [3,13].

Another crucial aspect of this study was the evaluation of inter-rater reliability between ChatGPT and the pediatric dentists. Cohen’s kappa statistics revealed almost perfect agreement between ChatGPT and two of the pediatric dentists, while a moderate agreement was observed with the other two clinicians. These findings suggest that ChatGPT can generate responses that are largely consistent with expert clinical judgment but may exhibit variability depending on the complexity of the case or the clinical decision-making. The variability in agreement could be attributed to differences in clinical reasoning, as human dentists may integrate subjective factors such as behavioral observations and individual patient history into their decision-making process-an element that AI lacks [14,15]. One of the key implications of this study is the potential role of AI in assisting dental professionals, particularly in settings where access to specialized pediatric dental care is limited. AI-powered diagnostic tools could serve as preliminary screening systems, guiding general practitioners or less experienced clinicians in recognizing common pediatric dental conditions and formulating initial treatment plans. Moreover, AI-based models could be used for patient education, helping parents understand their child’s dental condition, potential treatment options, and preventive strategies. Given that AI systems can provide instant, evidence-based responses, they may also be valuable for augmenting decision support in busy clinical environments [9].

Despite the promising results, several important considerations and limitations must be addressed before AI tools like ChatGPT can be confidently integrated into pediatric dental practice. First, while ChatGPT demonstrated a relatively high degree of diagnostic accuracy, it is essential to recognize that such AI models are fundamentally dependent on their pre-existing training datasets and algorithmic structure. They do not possess the ability to engage in direct patient interaction, conduct clinical examinations, or interpret non-verbal cues, which are the factors that are especially crucial in pediatric dentistry where patient cooperation, anxiety levels, and behavior significantly influence diagnosis and treatment decisions [3]. This inherent limitation means that AI may overlook clinically relevant variables that are evident only during in-person interactions, potentially reducing the reliability of its recommendations in real-world complex cases.

Additionally, ethical concerns play a central role in discussions surrounding the implementation of AI in clinical care. While AI-generated suggestions can be useful for educational and supportive purposes, they should never replace the nuanced clinical judgment of experienced professionals. There is a risk that over-reliance on AI, particularly in settings where practitioners may not be fully equipped to critically appraise its outputs, could result in suboptimal or inappropriate treatment choices. Clinical decisions often require context-specific insights that go beyond what text-based input can provide, especially in children with special health care needs, behavioral challenges, or socioeconomic complexities [13]. Therefore, AI tools must be deployed as adjunctive aids, with final decision-making resting firmly in the hands of trained clinicians [16].

Another limitation lies in the static nature of AI models like ChatGPT. These systems require continuous retraining and validation using updated datasets aligned with current clinical protocols, emerging research, and evolving treatment paradigms. Without this, there is a risk of AI providing outdated or inaccurate guidance that may not reflect best practices or current evidence-based standards. Furthermore, the “black-box” architecture of generative AI presents significant challenges in terms of transparency and accountability. Unlike human clinicians who can explain the rationale behind each diagnostic or therapeutic decision, AI models typically do not offer interpretability of their internal reasoning processes. This opacity can hinder clinician confidence, reduce adoption, and present legal or ethical challenges in cases where outcomes are questioned [17]. Enhancing explainability will be vital for building trust in AI tools and facilitating their integration into clinical workflows so that clinicians can trace how specific inputs influence outputs.

The pilot study has several limitations that must be acknowledged. The small sample size (n = 10), simulated case-based design, and absence of live patient interaction limit the generalizability of the findings. The study relied on standardized textual inputs, which do not reflect the variability and unpredictability encountered in daily clinical practice. Further large-scale studies using real-time patient encounters, diverse populations, and varying case complexities are necessary to evaluate the true clinical utility, safety, and acceptance of AI tools like ChatGPT in pediatric dentistry.

The results of this study provide a foundation for further research exploring the applications of AI in pediatric dentistry. Future studies should involve larger sample sizes and diverse patient populations to assess AI’s performance across different clinical scenarios. Additionally, longitudinal studies assessing AI’s role in long-term treatment planning and patient outcomes would provide more comprehensive insights into its clinical utility. Exploring hybrid models that combine AI-driven diagnostics with real-time clinician input may further enhance the effectiveness of AI-assisted decision-making.

## Conclusions

Within the limitations of this pilot study, ChatGPT demonstrated a level of diagnostic and treatment planning accuracy that was comparable to that of experienced pediatric dentists in standardized case scenarios. While these findings are encouraging, they should be interpreted with caution due to the small sample size and simulated nature of the cases. ChatGPT may have potential as a supportive tool in pediatric dental settings; however, further research involving larger, real-time clinical datasets and diverse patient populations is essential before drawing definitive conclusions about its clinical applicability.
